# Associations of intimate partner violence and reproductive coercion with contraceptive use in Uttar Pradesh, India: How associations differ across contraceptive methods

**DOI:** 10.1371/journal.pone.0241008

**Published:** 2020-10-16

**Authors:** Shweta Tomar, Nabamallika Dehingia, Arnab K. Dey, Dharmendra Chandurkar, Anita Raj, Jay G. Silverman

**Affiliations:** 1 Division of Global Public Health, Center on Gender Equity and Health, University of California, San Diego School of Medicine, La Jolla, CA, United States of America; 2 Joint Doctoral Program, San Diego State University/University of California San Diego, San Diego, CA, United States of America; 3 Sambodhi Research and Communications Pvt. Ltd., Noida, Uttar Pradesh, India; Purdue University, UNITED STATES

## Abstract

Intimate partner violence (IPV) and reproductive coercion (RC)–largely in the form of pressuring pregnancy—appear to contribute to low use of contraceptives in India; however, little is known about the extent to which these experiences differentially affect use of specific contraceptive methods. The current study assessed the association of IPV and RC with specific contraceptive methods (Intrauterine Devices [IUDs], pills, condoms) among a large population-based sample of currently married women (15–49 years, n = 1424) living in Uttar Pradesh. Outcomes variables included past year modern contraceptive use and type of contraceptive used. Primary independent variables included lifetime experience of RC by current husband or in-laws, and lifetime experiences of physical IPV and sexual IPV by current husband. Multivariate logistic regression models were developed to determine the effect of each form of abuse on women’s contraceptive use. Approximately 1 in 7 women (15.1%) reported experiencing RC from their current husband or in-laws ever in their lifetime, 37.4% reported experience of physical IPV and 8.3% reported experience of sexual IPV by their current husband ever in their lifetime. Women experiencing RC were less likely to use any modern contraceptive (AOR: 0.18; 95% CI: 0.9–0.36). Such women also less likely to report pill and condom use but were more likely to report IUD use. Neither form of IPV were associated with either overall or method specific contraceptive use. Study findings highlight that RC may influence contraceptive use differently based on type of contraceptive, with less detectable, female-controlled contraceptives such as IUD preferred in the context of women facing RC. Unfortunately, IUD uptake remains low in India. Increased access and support for use, particularly for women contending with RC, may be important for improving women’s control over contraceptive use and reducing unintended pregnancy.

## Introduction

Women and girls in India have over 48 million pregnancies per year, around half (48%) of which are reported to be unintended [1]. These unintended pregnancies are associated with reduced maternal healthcare utilization [2–4] and consequent poor maternal and child health outcomes [5, 6]. Lack of modern contraceptive (including contraceptive pills, intrauterine devices [IUD], injectable contraception, male condoms, subdermal implants, diaphragms, lactational amenorrhea, and emergency contraceptive pills) use is the proximal causal factor behind unintended pregnancies worldwide [7, 8]. A study conducted in 35 low- and middle- income countries (LMICs) found that use of modern contraceptives could prevent 5 million unintended pregnancies occurring across these countries annually [9]. The National Family Health Survey (NFHS) 2015–16 found that less than half (48%) of the married women of reproductive age in India were using any form of modern contraceptive at the time of survey [10]. Increasing the use of modern contraceptives among women is therefore an important step towards reducing unintended pregnancies and related negative health outcomes. Further, contraceptive use is an important part of the Sustainable Development Goal (SDG) including Goal 3 on guaranteeing good health and well-being for all and Goal 5 on promoting equality and empowerment of women and girls [11].

One key factor behind low rates of modern contraceptive use is women’s low reproductive autonomy i.e. power to decide and control contraceptive use, pregnancy, and childbearing [12]. Research has shown that reproductive coercion (RC; partners/husbands and in-laws limiting access to and use of contraceptives and pressuring women to become pregnant against their will) in India is more prevalent in the context of physical and sexual violence against women by their partners (intimate partner violence; IPV) [13]. Such Gender Based Violence (GBV) is highly prevalent in India with lifetime experience of physical and sexual IPV among ever married women reported at 30% and 6%, respectively [10] and lifetime experience of partner and/or in-laws perpetrated RC reported by 12% of ever married women [14].

While the association of IPV with contraceptive use has been studied extensively, the results from these studies have been found to be contradicting. In the Indian context, IPV has been found to be associated with decreased likelihood of modern contraceptive use [15, 16]. However, studies conducted in other settings have found that IPV and contraceptive use are positively associated [17]. Recent research has suggested that, considering the different method mix (i.e. different contraceptive methods used by women) available in different contexts, these contradicting findings may be due to IPV being associated with increased likelihood of female-controlled method use (eg. Injectables, IUD etc.) but decreased likelihood of male-controlled method use (eg. condoms) [15, 16]. Associations between RC and contraceptive use has been relatively less studied, particularly in LMIC settings [14]. One study conducted in India found that RC is negatively associated with overall contraceptive use [14], but no studies to date, in India or in any other LMIC context, have compared associations of both IPV and RC with method-specific contraceptive use. Addressing this gap in knowledge can help in identifying the type of contraceptive methods preferred by women facing IPV and RC.

The current study assesses the associations of use of different modern contraceptive methods with IPV and RC among a population-based sample of married women in Uttar Pradesh (UP), the most populous state is India. Building on a previous study on prevalence of RC and IPV and associations with overall contraceptive use [14], findings from the current study will fill an important knowledge gap regarding whether method mix differs for women facing IPV and RC as compared to those not reporting such abuse. This, in turn, may inform public health programs (e.g. on family planning counselling) to better assist women facing IPV and RC in the Indian context, as well as other LMIC settings. Models can be developed to provide counseling around covert (without telling partner or in-laws) use of contraceptives, a typical response of women coping with RC. Such models have already been successfully tested in high income context [18, 19].

## Methods

### Study design

Data for analysis were collected as part of an evaluation of Uttar Pradesh Technical Support Unit (UP-TSU), an intervention targeted at improving the quality of public health facilities and services in UP. The data used in current analyses were collected via a population-based survey conducted from August to October 2016 that included items on socio-economic characteristics, family planning practices, IPV and RC. The intervention did not focus on reducing IPV or RC among women and hence did not influence the objective of this study. Of all the districts (n = 75) in the state, the lowest 25 districts based on a composite index of health indicators were designated as High Priority Districts (HPDs) [14]. This index included maternal mortality ratio (MMR), percentage of institutional deliveries, infant mortality rate (IMR), percentage of children 12–23 months fully immunized, total fertility rate (TFR) and modern contraceptive prevalence rate (mCPR). A representative sample of currently married women of reproductive age (15 to 49 years) was drawn using multi-stage sampling design from 49 districts with an oversample from these 25 HPDs of UP. Further details of the research design has been described elsewhere [14]. The current cross-sectional analyses utilizes the sub-sample of women who were not sterilized or were not currently pregnant, and whose husbands were not sterilized at the time of the survey (n = 1424). Study protocols were reviewed and approved by the National Rural Health Mission of Uttar Pradesh, Public Health Service—Ethical Review Board (PHS-ERB)—an independent ethical review board, and the Health Ministry Screening Committee of the Indian Council for Medical Research. Informed written consent was obtained by study participants and interviews were conducted in private space with only the participant woman present.

### Measures

The outcome variables included past year modern contraceptive use and type of contraceptive method used in the same period. *Any past year modern contraceptive use* was defined as use of any form of modern contraception (including contraceptive pills, intrauterine devices [IUD], injectable contraception, male condoms, subdermal implants, diaphragms, lactational amenorrhea, and emergency contraceptive pills) in the 12 months preceding the survey. Type of contraception used was captured using a categorical variable with nine categories—no modern contraceptive method used and use of each of the eight forms of modern contraceptives specified above.

The primary independent variables in the analysis included lifetime experience of RC by current husband or in-laws, and lifetime experience of physical IPV and sexual IPV by current husband. Lifetime RC was captured using a composite index of eight equally weighted items relating to coercion or force used by the husband or in-laws to limit women’s reproductive autonomy. A woman was considered to have faced RC ever in her lifetime if she responded “yes” to any of the following eight items: *whether woman’s husband or in-laws ever stopped her from going or refused to give permission for her to go to a clinic or community health event to get family planning; destroyed*, *hidden*, *or taken a family planning method (such as pills) away from her; told her that they would abandon her if she tried to prevent or delay getting pregnant; told her that she would be beaten if she tried to prevent or delay getting pregnant; told her that it was against their religion or culture to use family planning; told her that women who use family planning do this so that they can have sex with other men; told her that she could not use family planning because she did not have any or enough sons; or not permitted her to use contraceptives*. Lifetime RC experience was dichotomized as yes or no based on ‘yes’ response to any of these eight items. This measure was based on a previously published measure of RC adapted for the Indian context and shown to have adequate reliability (Cronbach’s alpha = 0.73) [14].

Lifetime physical IPV was assessed through seven questions including whether the woman’s current husband had *ever slapped her*, *twisted her arm or pulled her hair; pushed her*, *shook her*, *or threw something at her; tried to choke or burn her; kicked her*, *dragged her*, *or beat her up; punched her with his fist or with something that could hurt her; or threatened or attacked her with a knife*, *gun*, *or any other weapon*. Lifetime physical IPV were dichotomized as yes or no based on ‘yes’ response to any of these seven items. Similarly, woman’s experience of sexual IPV was indicated by a positive response to *whether the woman’s current husband ever done any of the following*: *physically forced her to have sexual intercourse with him when she did not want to; physically forced her to perform other sexual acts she did not want to; used threats or other actions to make her perform sexual acts when she did not want to; forced her to do something sexual that she found degrading or humiliating; or she had sexual intercourse when she did not want to because she was afraid of what her husband might do if she refused*. Lifetime sexual IPV were dichotomized as yes or no based on ‘yes’ response to any of these items. Both physical IPV and sexual IPV assessments were drawn from the WHO Multi-country Study on Women’s Health and Domestic Violence [20].

Socio-demographic measures included caste, religion, household wealth status, woman’s literacy, husband’s education, woman’s age, woman’s age at first marriage, and birth parity. Households were categorized into three social categories using caste and religion, from most to least marginalized; they were 1) Non-Muslim Scheduled Caste or Scheduled Tribe (SC/ST), 2) Muslim, and 3) neither SC/ST nor Muslim. Household wealth status was assessed using Standard of Living Index (SLI) which is a proxy for the economic status of the household [21] and is widely used in national Demographic and Health Surveys, including the India NFHS [10]. The households were categorized into 4 groups ranging from poorest (1) to wealthiest (4) based on SLI scores of 0 to 25, 26 to 50, 51 to 75, and 76 to 100 (range 0–100). A woman was considered to be literate if she reported being able to both read and write in at least one language. Husband’s education was dichotomized based on whether or not he had completed primary school. The legal age of marriage for women in India (i.e. 18 years) was used as a cut-off to create two categories- women married at age below 18 years and women married at age of 18 years or above.

### Analysis

Association of demographics with each outcome variable (any past year modern contraceptive use and type of contraceptive used) and predictors (lifetime RC, lifetime physical and sexual IPV) was assessed using chi-square test. Specific contraceptive methods used by 5 or fewer participants were collapsed into “other modern methods”. This grouping was not subject to further analysis. Chi-square tests were also used to test associations between the predictors and outcome variables. Logistic regression adjusted for socio-demographic variables was used to further assess the relationship (adjusted odds ratio [aOR] and 95% Confidence Interval [CI]) between any past year modern contraceptive use and predictors. Multinomial logistic regression models described similar relationships (adjusted relative risk ratio [aRRR] and 95% CI) between predictors and type of contraceptive used. Multiple iterations of the multinomial model were performed with different base outcomes to test the risk ratio of each type of contraceptive relative to different base category. Multivariate logistic regression models (binomial for any modern contraceptive use and multinomial for type of method used) including all three predictors—RC, physical IPV, and sexual IPV along with demographics were developed to determine independent effect of each form of abuse on women’s contraceptive use. We tested covariates for multicollinearity prior to model construction. We found no collinearity, so all covariates were included in our models. Appropriate sample weights based on the multistage sampling procedure were used in all analyses. Data were analyzed using STATA 16.0 software (StataCorp, USA).

## Results

Approximately 1 in 4 (22.8%) women reported use of any modern contraceptive in the 12 months preceding the survey, with 6.7% reporting use of contraceptive pills, 1.4% reporting use of an IUD, and 13.3% reporting use of male condoms (Table 1). Women in higher wealth categories were more likely to report use of any modern method of contraception, specifically pills, as compared to those in lower wealth categories. Literate women were more likely than illiterate women to report use of any modern contraceptive, specifically male condoms. Women whose husbands had completed primary education (vs lower than primary) were more likely to report condom use. The proportion of women reporting IUD use was higher among women who got married as minors (less than 18 years) as compared to those who were married at 18 years of age or later (all p values<0.05).

**Table 1:** Frequencies of sample demographics by outcomes of interest.

	Total		Past year FP use-any			Pills			IUD			Condom		
	Unwtd. N	% (95% CI)	Unwtd. N	% (95% CI)	p-value	Unwtd. N	% (95% CI)	p-value	Unwtd. N	% (95% CI)	p-value	Unwtd. N	% (95% CI)	p-value
Total	1770		362	22.8% (19.9–25.9)		87	6.7% (5.2–8.5)		23	1.4% (0.7–2.8)		230	13.3% (10.9–16.1)	
Background characteristics														
Age														
15–19	4	0.6% (0.2–1.8)	0	0.00%	0.683	0	0.00%	0.675	0	0.00%	0.227	0	0.00%	0.213
20–24	192	11.5% (9.1–14.5)	47	21.8% (14.2–32.0)		9	4.0% (1.3–12.0)		4	0.8% (0.2–2.3)		30	15.7% (9.4–24.9)	
25–29	314	20.3% (17.2–23.9)	84	21.5% (15.9–28.2)		18	6.5% (3.7–11.1)		2	0.1% (0.0–0.4)		62	14.3% (9.5–21.0)	
30+	914	67.6% (64.0–71.0)	231	23.5% (19.8–27.7)		60	7.2% (5.3–9.8)		17	1.9% (0.9–4.0)		138	12.7% (9.9–16.2)	
Age at marriage														
<18	1264	87.4% (84.1–90.1)	321	22.7% (19.4–26.3)	0.906	77	6.6% (4.9–8.7)	0.785	22	1.6% (0.8–3.2)	0.006	201	13% (10.5–15.9)	0.13
18+	160	12.6% (9.9–15.9)	41	23.3% (15.1–34.2)		10	7.4% (3.4–15.1)		1	0.1% (0.0–1.0)		29	15.4% (8.7–26)	
Wealth Quintile														
1 (poorest)	302	19.3% (15.4–23.9)	66	20.9% (15.4–27.7)	0.049	17	6.3% (3.8–10.3)	0.01	6	1.4% (0.5–3.8)	0.259	37	9.0% (5.4–14.8	0.142
2	557	37.2% (32.2–42.5)	128	19.6% (15.6–23.9)		40	5.7% (3.6–8.8)		6	0.7% (0.3–1.8)		70	11.9% (8.6–15.8)	
3	466	34.9% (30.2–39.9)	135	24.1% (18.3–31.1)		19	5.4% (3.2–9.1)		11	2.4% (0.9–6.5)		101	16.0% (11.9–21.1)	
4 (wealthiest)	99	8.6% (6.1–12.0)	33	35.1% (25.2–46.5)		11	16.8% (8.9–29.5)		0	0.00%		22	18.3% (9.5–32.4)	
Literacy														
Illiterate	957	66.2% (62.5–69.6)	218	20.2% (16.9–24.0)	0.025	56	6.1% (4.4–8.4)	0.423	13	1.5% (0.6–3.7)	0.679	131	10.7% (8.1–14.0)	0.0083
Literate	467	33.8% (30.4–37.5)	144	27.8% (22.4–33.9)		31	7.8% (4.9–12.2)		10	1.2% (0.5–2.5)		99	18.4% (13.9–24.0)	
Spouse literacy														
Illiterate	469	33.4% (29.2–37.8)	99	20.0% (16.0–24.9)	0.195	27	6.0% (3.7–9.5)	0.609	7	1.4% (0.5–4.4)	0.937	56	10.3% (7.0–14.9)	0.012
Literate	955	66.6% (62.3–70.8)	263	24.1% (20.3–28.4)		60	7.0% (5.1–9.5)		16	1.4% (0.5–3.4)		174	14.8% (11.9–18.3)	
Caste/religion														
Neither SC/ST nor Muslim	847	58.2% (53.2–63.1)	233	24.6% (20.5–29.3)	0.197	62	7.6% (5.5–10.5)	0.424	16	1.7% (0.7–4.1)	0.513	142	14.6% (11.2–18.7)	0.154
SC/ST	320	21.8% (18.0–26.2)	79	22.6% (17.2–29.0)		16	5.8% (3.4–9.9)		4	0.9% (0.3–3.2)		54	14.4% (10.6–19.4)	
Muslim	257	20.0% (15.3–25.7)	50	17.5% (12.3–24.3)		9	4.8% (2.2–10.0)		3	0.8% (0.2–3.4)		34	8.4% (5.1–13.7)	
Parity														
0	211	14.9% (12.0–18.4)	53	22.9% (15.4–32.5)	0.541	12	5.7% (2.8–11.6)	0.876	4	0.9% (0.2–3.6)	0.175	35	15.9% (9.6–25.1)	0.022
1	148	9.2% (7.0–11.8)	43	28.3% (19.6–38.9)		12	7.0% (3.1–15.3)		1	0.2% (0.0–1.2)		29	20.2% (12.1–31.7)	
2	229	14.9% (12.6–17.5)	63	25.3% (17.8–34.7)		7	5.2% (1.8–14.0)		4	0.8% (0.2–2.4)		49	19.0% (12.7–27.5)	
3+	836	61.1% (57.4–64.6)	203	21.3% (17.8–25.2)		56	7.2% (5.2–9.8)		14	1.8% (0.8–4.2)		117	10.3% (7.8–13.4)	

Approximately 1 in 7 women (15.1%) reported ever experiencing RC from their current husband or in-laws (Table 2). More than 1 in 3 women (37.4%) reported ever experiencing physical IPV and 8.3% reported ever experiencing sexual IPV from their current husband.

**Table 2:** Prevalence of reproductive coercion, husband IPV and in-law IPV.

	No		Yes	
	Unwtd. N	% (95% CI)	Unwtd. N	% (95% CI)
Reproductive coercion	1218	84.9% (80.9–88.1)	206	15.1% (11.9–19.1)
Husband physical IPV	887	62.6% (56.8–68.0)	537	37.4% (32.0–43.2)
Husband sexual IPV	1302	91.8% (88.6–94.1)	122	8.3% (5.9–11.4)

RC was found to be associated with overall modern method of contraception. In logistic models (binomial and multinomial with different base outcome) adjusted for demographics (Table 3), women who had experienced RC were less likely to report use of any modern contraceptive (aOR 0.18; 95% CI, 0.09–0.36) in past 1 year. Findings from multinomial models indicate that women who face RC were less likely to use pills as compared to using no method (aRRR 0.02; 95% CI, 0.00–0.07). In the model with pills as base outcome, women who faced RC were more likely to use IUDs than pills (aRRR 63.74; 95% CI, 7.42–547.20) and condoms than pills (aRRR 13.9; 95% CI, 2.89–67.30). Neither physical nor sexual IPV by current husband were associated with any past year modern contraceptive use or type of contraceptive used.

**Table 3:** Logistic (binomial and multinomial) adjusted for demographics.

			Method used vs no contraceptive use			Method wise comparison		
		Any modern method vs no modern method	Pills vs no modern method	IUD vs no modern method	Condom vs no modern method	IUD vs Pills	Condom vs Pills	Condom vs IUD
		aOR (95% CI)	aRRR (95% CI)	aRRR (95% CI)	aRRR (95% CI)	aRRR (95% CI)	aRRR (95% CI)	aRRR (95% CI)
Reproductive coercion	No	Ref	Ref	Ref	Ref	Ref	Ref	Ref
	Yes	0.18*** (0.09–0.36)	0.02*** (0.00–0.07)	1.07 (0.21–5.46)	0.23 (0.12–0.47)	63.74*** (7.42–547.20)	13.92*** (2.89–67.30)	0.22* (0.04–1.32)
Husband physical IPV	No	Ref	Ref	Ref	Ref	Ref	Ref	Ref
	Yes	0.72* (0.51–1.02)	0.83 (0.43–1.59)	0.72 (0.15–3.39)	0.67* (0.43–1.02)	0.87 (0.16–4.63)	0.81 (0.37–1.74)	0.93 (0.18–4.85)
Husband sexual IPV	No	Ref	Ref	Ref	Ref	Ref	Ref	Ref
	Yes	0.62 (0.33–1.16)	0.61 (0.25–1.51)	0.32 (0.04–2.51)	0.69 (0.32–1.50)	0.52 (0.06–4.53)	1.14 (0.38–3.38)	2.17 (0.28–16.66)

* Trend towards statistical significance (p <0.1)

*** Statistically significant at p <0.01

Findings from the multivariate models adjusted for both demographics and inclusive of all three forms of abuse (Table 4) were identical, with women reporting lifetime experience of RC less likely to use any modern contraceptive (aOR 0.19; 95% CI, 0.10–0.38), less likely to use pills than using no method (aRRR 0.02; 95% CI, 0.00–0.07) more likely to use an IUD than pills (aRRR 69.41; 95% CI, 9.35–515.45) and more likely to use a condom than pills (15.00; 95% CI, 3.01–74.79). Additionally, women who faced RC were less likely to use a condom as compared to no contraceptive use (aRRR 0.24; 95% CI, 0.12–0.49) in the models inclusive of all forms of violence.

**Table 4:** Multivariate logistic (binomial and multinomial) adjusted for demographics and inclusive of different forms of violence.

			Method used vs no contraceptive use			Method wise comparison		
		Any modern method vs no modern method	Pills vs no modern method	IUD vs no modern method	Condom vs no modern method	IUD vs Pills	Condom vs Pills	Condom vs IUD
		aOR (95% CI)	aRRR (95% CI)	aRRR (95% CI)	aRRR (95% CI)	aRRR (95% CI)	aRRR (95% CI)	aRRR (95% CI)
Reproductive coercion	No	Ref	Ref	Ref	Ref	Ref	Ref	Ref
	Yes	0.19*** (0.10–0.38)	0.02*** (0.00–0.07)	1.12 (0.27–4.68)	0.24*** (0.12–0.49)	69.41*** (9.35–515.45)	15.00*** (3.01–74.79)	0.22* (0.04–1.05)
Husband physical IPV	No	Ref	Ref	Ref	Ref	Ref	Ref	Ref
	Yes	0.86 (0.60–1.23)	1.08 (0.53–2.18)	0.80 (0.18–3.56)	0.76 (0.50–1.17)	0.74 (0.15–3.69)	0.71 (0.31–1.60)	0.96 (0.20–4.66)
Husband sexual IPV	No	Ref	Ref	Ref	Ref	Ref	Ref	Ref
	Yes	0.64 (0.32–1.24)	0.52 (0.20–1.41)	0.37 (0.03–4.21)	0.76 (0.33–1.74)	0.70 (0.05–9.15)	1.45 (0.46–4.60)	2.08 (0.18–24.03)

* Trend towards statistical significance (p <0.1)

*** Statistically significant at p <0.01

## Discussion

More than 1 in 7 (15%) women are affected by RC in this population-based sample of ever married women. As discussed earlier, previous study in UP reported an RC prevalence rate of 12% among ever-married women in reproductive age [14]. The current study was based on the same sample but excluded women who may not need contraceptives at the time of study i.e. women who were sterilized, whose husbands were sterilized and who were pregnant at the time of study. Thus, RC appears to be pervasive among women in LMIC settings, especially among women who may have a need for contraceptives.

Among women experiencing RC, less than 1 in 15 (6.4%) reported use of any modern contraceptive in the last 1 year; similar to previous research [14], these women were significantly less likely to use modern contraceptives as compared to women who had not experienced RC, after adjusting for effect of IPV and demographics. Similar to the earlier study among this population, overall contraceptive use was not found to be associated with IPV. These findings show that, similar to other contexts [22–24], RC among women in Uttar Pradesh reduces their ability to successfully use modern contraceptive method.

The model for method specific contraceptive use shows that experience of RC was associated with decreased likelihood of pill and condom use but not IUD. The findings on reduced condom use are consistent with previous studies in India demonstrating that men perpetrating IPV are less likely to use condoms [25–27]. However, the finding on reduced likelihood of pill use based on RC would appear to contradict the hypothesis that female-controlled methods will be positively associated with forms of GBV, as pills are typically considered as female-controlled contraceptives that can be used without the knowledge of male partners. However, a recent study on GBV and contraceptive methods posited that contraceptive pill use should only be considered truly female-controlled in cases where it is feasible for women to both conceal the pill package, and to take the pill unobserved [28]. This is likely not the case in this setting, as this study was conducted in rural areas of Uttar Pradesh where the average household size is over five, approximately half (44%) of households include women’s in-laws [10], and women generally live in close-quarters with other family members, with each room occupied by an average of three family members [29]. This close proximity to other family members may make it difficult for women facing RC to conceal and use pills without knowledge of husband or in-laws, making pills use less likely in cases of RC.

In contrast, experience of RC increased the likelihood of IUD usage, both in comparison to pills and condoms. Earlier research has also found that women find IUDs easier to use covertly than the oral contraceptive pills [30]. While insertion of IUD is a one-time activity which can be done when husband or in-laws are not around, pills need to be consumed regularly and therefore, involve a higher risk of detection. However, the results from NFHS, 2015 show that only 1.5% women in India and 1.2% in Uttar Pradesh use IUD [10]. One possible reason behind this low IUD use is the lack of commitment from national and state government to take steps required for increasing awareness of and access to IUDs [31]. This may be a partial explanation for the most commonly used method among women facing RC being condoms. However, this does not explain the increased likelihood of condom use as compared to pills among women facing RC. A context-specific explanation for greater use of condoms as compared to pills is perpetration of such coercion by family members other than husband. As reported in the previous study, in-laws were the sole perpetrator in almost half of all RC cases [14] in this sample. For women facing RC by in-laws but not by husband, i.e., where husbands are not attempting to coerce her to become pregnant, condom use may well be the most feasible method of contraception. Future studies investigating the connection of use if specific methods based on source of RC will be important to clarify these findings. While the current study collected data on perpetrator of RC, multivariate analysis for different RC perpetrators could not be conducted due to small cell sizes (small strata), particularly regarding IUD use.

The current results add to the building consensus that screening for RC should be included in confidential contraceptive counselling, as this experience appears to be a significant factor in women’s choices and needs regarding type of contraceptive methods. Enabling women facing RC to successfully use contraceptives can help in reducing unintended pregnancies [14, 32] and related adverse health consequences [5, 6, 14, 33]. Models of clinic-based intervention found to successfully address RC and to reduce pregnancy among women seeking contraceptives [18, 19] should be considered for adaptation and implementation in rural UP and similar contexts. Since IUD prevalence is very low in India and the current study found it to be a preferred method of contraception among women facing RC, improving awareness about and access to IUDs by the Indian government may both increase overall contraceptive use in the country, and also better help women to preserve their reproductive autonomy.

## Limitations

While the current findings provide important insights into the type of contraceptives used by women facing RC, there are several limitations related to the study design that are worth noting. The data used include lifetime RC and IPV but past 12 months contraceptive use. Data to assess current desire to become pregnant, an important factor behind contraceptive use among women, was not collected. The study also has limitations in the sample size within the strata of types of contraceptive which yields small cell sizes, making it difficult to detect differences by group. This is reflected in the large confidence intervals obtained for use of IUDs which have a very low prevalence. A larger sample and greater representation across contraceptive types may present clearer findings. As contraceptive use and particularly IUDs and other less commonly used forms of contraceptive use increase under FP2020 activities, further research could help provide greater clarity into these issues. Also, we interpret study findings to indicate that the positive association between RC and IUD use may be because of women’s control and potentially even the possibility of women’s covert use of this method. Further research should assess the quantitative association between RC and covert contraceptive use more directly and can also delve into the qualitative reasons for type of contraceptive used among women contending with RC to provide more insight into these findings. Data used in the study is cross-sectional and therefore causality cannot be inferred. Lastly, the study used self-reported data which might have introduced social desirability and recall bias.

## Conclusion

Reproductive coercion is prevalent and critical barrier to overall use of contraceptive methods among women in Uttar Pradesh. Among women facing RC, IUD is the most preferred contraceptive method, and pills, the most commonly used female controlled method used in India, may be more difficult to use for these women. Although, further research is warranted, these findings suggest that confidential identification of women facing RC by community health workers and health facility-based providers may result in increased contraceptive use, especially as new female-controlled contraceptives (injectables and implants) are currently being introduced in UP and other Indian states.
